# Error in Figure 2

**DOI:** 10.1001/jamanetworkopen.2019.5248

**Published:** 2019-05-17

In the Original Investigation titled “Assessment of Health Care Utilization and Cost of Targeted Drug Delivery and Conventional Medical Management vs Conventional Medical Management Alone for Patients With Cancer-Related Pain,”^1^ published April 5, 2019, in Figure 2, the graphical representation of all-cause costs for the conventional medical management only group for 12 months was missing. This article has been corrected.^1^
